# Editorial: What makes us human: from genes to machine

**DOI:** 10.3389/fnins.2025.1682082

**Published:** 2025-10-15

**Authors:** Idan Segev

**Affiliations:** The Edmond and Lily Safra Center for Brain Sciences, The Hebrew University of Jerusalem, Jerusalem, Israel

**Keywords:** human uniqueness, brain and creativity, language and consciousness, multiscale neuroscience, human neuron computation, ethical challenges of non-biological minds, evolutionary neurobiology

## Introduction

What makes us human—our boundless creativity, symbolic language, conscious self-awareness, and our capacity for science and art? This timeless question has captivated scientists for millennia. Yet in recent years, with rapid technological and conceptual advances—from genes to machines—the scientific quest to understand human uniqueness has gained new urgency and depth.

This Frontiers Research Topic brings together multidisciplinary contributions that illuminate humanity through a multiscale lens—spanning genes to neurons, circuits to whole-brain connectome topology, and biological evolution to computational approaches for brain function. Collectively, these studies reveal that human distinctiveness is not rooted in a single trait, but emerges from the dynamic interaction of multiple systems: intricate cortical microcircuits and cultural scaffolding, evolutionary pressures and neural computations, spontaneous brain dynamics and social learning.

The articles show how the fine-grained structure and biophysical complexity of human pyramidal neurons enhance their computational power. They trace evolutionary shifts in motor pathways and neurodevelopmental genes and suggesting that a universal neuronal mechanism underlying human creativity consists of spontaneous, ultra-slow, fluctuations of activity in our brains. Others studies explore how language, consciousness, and intelligence may arise not only in biological organisms, but in non-traditional systems—raising new ethical and profound conceptual challenges.

From the red nucleus to resting-state networks, from paleogenomic signatures to the socio-cultural reprogramming of the mind, this Research Topic offers a deeply integrative view of what makes us human. It invites us to think across disciplines and scales—and, perhaps most importantly, to imagine what lies ahead in our evolving story.

## Article highlights

Faskowitz et al., systematically compare the topology of anatomical brain networks across mammals to investigate their evolutionary and taxonomic relationships. Using the MaMI database—a collection of *ex vivo* diffusion MRI-derived brain networks covering 125 species across 12 mammalian orders—the authors calculate diverse network distance metrics to quantify dissimilarity between species. They demonstrate that species within the same taxonomic order have significantly more similar connectome topology than those in different orders, and that network distances positively correlate with phylogenetic distances derived from genetic data (based on analysis across 10,000 plausible phylogenies). The findings support the idea that brain wiring patterns embody taxonomic and phylogenetic signatures across mammalian evolution.

Boeckx hypothesizes that the human condition is best understood not through narratives of a sudden “cognitive modernity” nor through the assumption of complete cognitive similarity with Neanderthals and Denisovans. Instead, he emphasizes paleogenomic signals—notably genomic regions devoid of introgression and under positive selection—as pointing to mutations affecting neurodevelopment and temperament, shaping cultural trajectories in specific ways. These genomic changes likely influenced shifts in learning styles and language phenotypes, altering what we learn, how symbols combine, and how community size and configurations evolve. The authors claims that our communicative uniqueness stems less from a dedicated “language module” and more from socially mediated innovation, built on reduced reactive aggression and enhanced cooperative sociability. The paper claims that human symbolic communication is the outcome of intertwined biological, behavioral, and cultural evolution, portraying us as true “hunter-gatherers of words.”

Rockland outlines the neuroanatomical specializations that distinguish humans (and non-human primates) from other mammals, emphasizing a multiscale comparative framework that spans from subcellular to macrostructural levels. She highlights key human-relevant traits such as thicker supragranular cortical layers, reduced neuron density paired with increased synaptic complexity, and more diversified and elaborated astrocytes and glial cells, all suggesting enhanced neural integration and processing capacity. At the subcellular scale, humans exhibit larger synaptic active zones, greater vesicle pools, and more intimate astrocytic ensheathment—features linked to synaptic efficiency. The article also discusses hemispheric asymmetries in relation to uniquely human functions such as language and fine motor control. Rockland concludes by advocating for a unified, systems-level neuroanatomy that integrates cellular, molecular, and connectivity data to better understand what makes us human.

Deverett argues that with advances in artificial intelligence and emergence of novel forms of awareness—whether through machines, brain-computer interfaces, or potential extraterrestrial life—our ability to recognize, assess, and ethically interact with non-traditional consciousness becomes increasingly urgent. In this provocative article, Deverett draws on the unique lens of anesthesiology, a field devoted to modulating human consciousness, to propose a framework for engaging with unfamiliar minds. By distinguishing between levels and contents of consciousness, and acknowledging the philosophical and practical limits of current models, the paper suggests that anesthetic tools—such as behavioral inference, monitoring, and ethical deliberation—may serve as vital guides. Ultimately, the article urges us to prepare for a future in which we may need to gently dim, or protect, consciousness in entities vastly unlike ourselves.

Malach conjectures that a universal neuronal mechanism underlying human creativity consists of spontaneous, ultra-slow, activity fluctuations (also called resting-state activity). These intrinsic neural dynamics, rather than external stimuli, generate the cognitive seeds of innovation by integrating internal “noise” with learned, deterministic information. He defines creativity as the generation of ideas that are both novel and meaningful, setting a high bar beyond mere randomness. Malach proposes a “template-matching” strategy in searching for neuronal mechanisms of creativity: identifying central characteristic neural signatures that are common across the entire spectrum of creative acts, then linking them experimentally to creative behavior. Empirical studies correlating resting-state activity with verbal creativity tasks across different individuals support this model. Computational simulations further illustrate how the interplay between stochastic-deterministic brain activities optimizes the search and thus enables original yet contextually-grounded discovery of creative solutions. Overall, the review suggests that the mysterious spark of creativity may arise from the semi-ordered chaos of background brain activity fused with the structure of expertise and learning.

Żuromski and Pacholik-Żuromska proposes that our cognition is not just a product of our brains, but of the cultural tools and social environments that have reprogrammed it over time. Drawing from evolutionary biology, neuroscience, and cognitive science, the authors argue that the true leap in human cognition did not come from brain size alone, but from our unparalleled ability to integrate artifacts—tools, language, and technology—into our cognitive and bodily systems. These “cognitive gadgets,” shaped by cultural evolution, have restructured our brains' architecture and extended our thinking far beyond the skull. From the body-augmenting role of exoskeletons to the mind-transforming power of language, humans stand apart as a species whose mental abilities are built through collaboration, shared symbols, and generations of accumulated knowledge. Ultimately, this socio-cultural reprogramming has turned us into “ultra-social” beings whose intelligence is collective, cumulative, and ever-evolving.

Stacho et al. uncovers a striking evolutionary shift in the architecture of the red nucleus (RN)—a brainstem structure central to motor control. Under their openly-available BigBrain model, they created high-resolution cytoarchitectonic delineations of the human red nucleus to capture inter-subject variations in quantitative terms. By mapping and comparing the red nucleus in 20 primate species, from lemurs to humans, the researchers reveal that the magnocellular component (RNm), which drives coarse motor output via the rubrospinal tract, has dramatically shrunk in apes and humans. In contrast, the parvocellular component (RNp), which is part of the olivo-cerebellar circuitry, scales consistently with brain size and becomes prominent in human. This suggests a transition, during primate evolution, from spinal-centric motor control toward refined, cerebellar-integrated coordination—likely supporting fine hand movements and sensorimotor sophistication. The team also mapped individual variability in RN structure, offering new tools for future neuroscience research. Together, these findings illuminate how ancient motor systems are restructured across evolution, providing insight into the neural foundations of dexterity, tool use, and human cognitive uniqueness.

Gidon et al. challenge the idea that consciousness simply arises from the right pattern of neural computations. They explore this by simulating a visual task in a brain-inspired network, and recording how each artificial neuron responds. Then, in a twist, they replay the exact same neural signals back into the system—but in a way that removes “counterfactual activity”: the system no longer has the capacity to respond differently if the input or context were to change. In other words, while the visible neural activity looks identical, the network has been stripped of its ability to compute meaningful alternatives—it has lost its “what if” potential. Remarkably, even though the ongoing network activity unfolds naturally, the underlying computation loses its richness. This reveals a critical insight: computation depends not just on what the brain does, but on what it could do. This challenges the view that consciousness arises simply from computation, as it implies that eliminating counterfactuals undermines the very basis of experiential states—even when overt neural dynamics appear intact.

Shapira et al. present key theoretical insights from a decade of developing detailed biophysical models of human layer 2/3 cortical pyramidal neurons, grounded in high-resolution morpho-electrophysiological data from neurosurgical samples. These models reveal that the disproportionately large dendritic tree of these neurons imposes large input impedance imposed by the large dendritic tree on the soma together with the enriched ion channls in the dendrites endows human L2/3 pyramidal neurons with exceptional signaling capabilities: sharply rising (“kinky”) somatic spikes that reliably track high-frequency inputs, rapid dendritic excitatory post-synaptic potentials (EPSPs) propagation, and pronounced dendritic compartmentalization—supporting parallel non-linear processing. Complementary machine learning/deep learning (DNN) approaches show that replicating the input/output properties of human L2/3 pyramidal neurons requires significantly deeper DNNs than for rodent neurons, underscoring the computational complexity of human pyramidal neurons. These distinctive features may underpin advanced human cognitive capacities. Looking ahead, the authors advocate for expanding modeling to diverse human cell types and integrating dense EM-based reconstructions to probe circuit-level dynamics of the human cortex.

